# Template-Free Morphology Engineering of CeO_2_ for Dye-Wastewater Purification: From Porous Architectures to Adsorption-Assisted Photocatalytic Removal

**DOI:** 10.3390/molecules31081244

**Published:** 2026-04-09

**Authors:** Yaohui Xu, Quanhui Hou, Liangjuan Gao, Zhao Ding

**Affiliations:** 1 Laboratory for Functional Materials, School of New Energy Materials and Chemistry, Leshan Normal University, Leshan 614000, China; xyh1986@lsnu.edu.cn; 2 Leshan West Silicon Materials Photovoltaic and New Energy Industry Technology Research Institute, Leshan 614000, China; 3 School of Automotive Engineering, Yancheng Institute of Technology, Yancheng 224051, China; hqhdyx66@ycit.edu.cn; 4 College of Materials Science and Engineering, Sichuan University, Chengdu 610065, China; lgao87@scu.edu.cn; 5 College of Materials Science and Engineering, National Engineering Research Center for Magnesium Alloys, National Innovation Centre for Industry-Education Integration of Energy Storage Technology, Chongqing University, Chongqing 400044, China

**Keywords:** CeO_2_, template-free synthesis, morphology engineering, dye-wastewater purification, adsorption, adsorption–photocatalysis coupling

## Abstract

Cerium dioxide (CeO_2_) has emerged as a structurally versatile oxide for dye-wastewater purification because its architecture, porosity, and surface accessibility can be tuned over a wide range while maintaining good chemical stability and environmental compatibility. Recent studies show that template-free or low-template routes can generate porous, mesoporous, multilayered, and flower-like CeO_2_ architectures with rapid dye uptake and, in some systems, adsorption-assisted photocatalytic removal. However, CeO_2_-based dye removal has often been discussed either within broad surveys of environmental applications or from composition-centered viewpoints, whereas the more fundamental question is how synthesis route controls architecture formation and how architecture, in turn, governs adsorption and subsequent removal behavior. This mini-review addresses that question from a morphology-centered perspective. It first examines template-free and low-template routes for constructing structured CeO_2_, then discusses how porosity, hierarchical assembly, and surface accessibility regulate adsorption kinetics and equilibrium capacity in dye-containing aqueous systems. It further considers adsorption-assisted photocatalytic removal and argues that dark adsorption should be regarded as the structural first step rather than a secondary contribution. On this basis, the review shows that rare-earth doping in these systems is most usefully understood as a secondary tuning strategy that refines an already favorable host architecture by modifying surface interaction, optical response, or reactive-species generation. Overall, the available evidence indicates that CeO_2_-based dye-wastewater purification is most meaningfully interpreted through a route–architecture–function framework in which morphology defines the host, adsorption organizes the local reaction environment, and doping serves mainly as structure-assisted tuning. This perspective shifts the design logic of CeO_2_ from empirical performance optimization toward rational structure-directed construction of integrated removal platforms.

## 1. Introduction

Dye-containing wastewater remains a persistent environmental challenge because synthetic dyes are produced and consumed on a large scale, while a measurable fraction is ultimately discharged into water streams during textile dyeing, printing, papermaking, leather processing, and related industrial operations [1]. Among these pollutants, azo dyes are particularly problematic because of their structural stability, poor biodegradability, and potential toxicity after environmental transformation [2,3,4,5]. For this reason, dye removal has long relied on multiple physical, chemical, and biological approaches, including adsorption, membrane separation, coagulation–flocculation, advanced oxidation, and photocatalytic degradation [6,7,8]. Yet, in practical treatment, adsorption continues to occupy a central position because of its operational simplicity, tolerance toward toxicants, and relative ease of implementation [9].

Against this background, the key question is no longer whether dyes can be removed but what kind of material can combine fast uptake, sufficient capacity, structural robustness, and practical regenerability. Traditional porous adsorbents such as activated carbon, zeolites, alumina-based materials, and natural clays have been widely used, but they often face limits in terms of selectivity, adsorption kinetics, regeneration, or cost once higher performance is required [10]. This has stimulated growing interest in metal-oxide-based adsorbents and adsorptive photocatalysts, particularly those with controllable pore structure, exposed surface sites, and architecture-dependent mass-transfer behavior [11,12]. Within this broader materials space, CeO_2_ is especially attractive because it combines environmental compatibility, thermal and chemical stability, and a structurally flexible fluorite framework that can be shaped into a wide range of nano- and microarchitectures [13,14,15].

CeO_2_ is, of course, better known in catalysis and energy research for its redox chemistry, oxygen vacancies, and oxygen-storage behavior. Broad reviews have therefore naturally emphasized catalytic conversion, defect engineering, and multifunctional applications at a general level. However, once the target shifts from gas-phase catalysis or oxygen buffering to dye-wastewater purification, a different design axis becomes more important. In this context, the decisive issue is often not defect chemistry alone, but how morphology and pore architecture regulate surface accessibility, diffusion path length, adsorption–desorption equilibration, and, in some systems, the local enrichment of dye molecules prior to photocatalytic transformation. In other words, the removal behavior of CeO_2_ in aqueous dye systems is strongly morphology-dependent, and this dependence merits discussion in its own right rather than being absorbed into a broad survey of CeO_2_ applications [16,17].

This morphology-centered perspective has become increasingly relevant because recent work has shown that CeO_2_ can be directed into porous, mesoporous, multilayered, and flower-like architectures with markedly different dye-removal behavior. More importantly, these structures do not merely differ in appearance; they differ in accessible porosity, transport pathways, surface-site exposure, and their ability to support either direct adsorption or adsorption-assisted photocatalytic removal. This point suggests that the functional behavior of CeO_2_ in wastewater purification is best interpreted not from composition alone, but from a route–architecture–function sequence in which synthesis determines structure and structure, in turn, conditions removal performance.

A second reason why this topic warrants a dedicated mini-review is that morphology control in CeO_2_ is no longer limited to conventional surfactant- or template-assisted synthesis. A growing body of literature now shows that template-free or low-template routes can generate porous, mesoporous, hollow, rod-like, octahedral, and flower-like CeO_2_ architectures through relatively simple hydrothermal, solvothermal, precipitation-assisted, or oxidation-assisted processes [18,19]. This is not a trivial synthetic detail. Heavy reliance on organic templates and structure-directing agents often increases process complexity, cost, and post-treatment burden, whereas template-free routes make morphology engineering more practical and more relevant to environmental applications. At the same time, they offer a cleaner opportunity to examine how architecture itself influences dye uptake, rather than conflating morphology effects with residual template chemistry or complicated multicomponent formulations.

**Figure 1:**
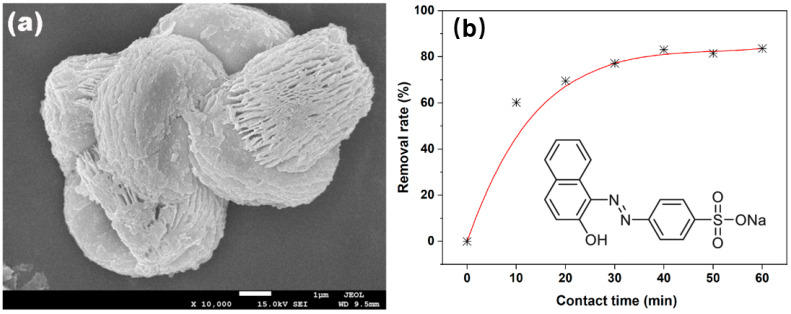
A flower-like hierarchical CeO_2_ host as a morphology-level entry point for dye removal. Panel (**a**) shows the multilayered interwoven morphology of undoped CeO_2_, while panel (**b**) shows the time-dependent adsorption profile of Acid Orange 7 (AO7) on the same undoped host (‘*’ here denotes the sampling points collected every 10 min from 0 to 60 min.), with the structural formula of AO7 included as an inset to clarify its aromatic framework. Together, these panels introduce the central logic of this review: synthesis route constructs host architecture, and architecture in turn governs adsorption and subsequent removal behavior. Adapted from Ref. [20].

The conceptual framework of the present review is introduced in Figure 1. Panel (a) shows that undoped CeO_2_ can already form a flower-like hierarchical host composed of interwoven nanosheet subunits, while panel (b) shows that the same host exhibits rapid initial Acid Orange 7 (AO7) uptake followed by a slower approach to adsorption–desorption equilibrium. To improve structural readability, the aromatic framework of AO7 is also shown directly in panel (b) as an inset. Read together, these two panels introduce the central logic of this review: the route used to construct the CeO_2_ host determines the resulting architecture, and that architecture in turn governs the accessibility, transport behavior, and early-stage adsorption response of the system. In this sense, Figure 1 serves as a morphology-level entry point to the route–architecture–function framework developed in the following sections, rather than as a summary of all CeO_2_ architectures discussed later [20].

This framework should not be understood as a rejection of established concepts such as structure–activity relationships, adsorption–photocatalysis coupling, or Langmuir–Hinshelwood-type surface-reaction models. Rather, its purpose is to place these commonly discussed ideas into a more explicit order of interpretation for aqueous dye-removal systems. In the present view, synthesis route first constructs the CeO_2_ host, host architecture then governs accessibility, transport, and local dye enrichment, and only on that basis do optical response, defect chemistry, and compositional tuning become functionally decisive. The perspective advanced here is therefore not that CeO_2_ obeys fundamentally new physicochemical principles, but that known behaviors can be organized into a design-oriented hierarchy that is more directly useful for morphology-engineered dye-wastewater purification. In this sense, the present framework is related to, but distinct from, broad structure–activity discussions in ceria nanochemistry and from studies in which adsorption and degradation are already treated as coupled surface events during AO7 removal [7,16]. Whereas those viewpoints usually emphasize either surface reactivity itself or adsorption–degradation coupling as an operational sequence, the present perspective places synthesis route and host architecture at the beginning of the interpretive hierarchy.

Accordingly, the purpose of this mini-review is not to catalog all environmental uses of CeO_2_ but to discuss dye-wastewater purification from the standpoint of morphology engineering. The discussion begins with template-free and low-template routes for constructing porous and hierarchically assembled CeO_2_ architectures. It then turns to morphology–adsorption relationships, focusing on how porosity, hierarchy, and structural accessibility influence dye uptake kinetics and equilibrium capacity. A subsequent section examines the transition from pure adsorption to coupled adsorption–photocatalytic removal, with Acid Orange 7 as a representative probe system. On this basis, the review finally considers how rare-earth doping can act as a secondary tuning variable within a morphology-dominated design framework and argues that future progress in CeO_2_-based wastewater purification will depend increasingly on rational structure-directed design rather than on isolated performance optimization.

## 2. Template-Free and Low-Template Routes to Structured CeO_2_

The recent development of CeO_2_-based materials for dye-wastewater purification has shown that synthesis route is not merely a preparative detail, but the first structural variable that determines how adsorption and subsequent removal behavior can be realized. Hydrothermal and solvothermal methods are especially powerful in this regard because they allow the morphology, size, and assembly mode of CeO_2_ to be tuned through solvent composition, precursor chemistry, temperature, and reaction time, thereby giving access to spindle-like, octahedral, spherical, flower-like, and mesoporous structures [13,17,18,19,20]. More importantly for the present review, these routes can often be implemented without heavy reliance on hard or soft templates, which makes them especially relevant to environmentally oriented applications where simplicity, scalability, and reduced post-treatment burden are important.

Template-free and low-template strategies deserve special attention because they show that complex CeO_2_ architectures do not necessarily require elaborate structure-directing agents. In route-controlled CeO_2_ systems, structurally differentiated oxide frameworks can emerge from comparatively simple hydrothermal or solvothermal environments through precursor hydrolysis, local aggregation, oriented growth, and post-calcination reorganization [17,18,19,20,21,22]. This point is important because it shifts the emphasis away from template chemistry itself and toward the more fundamental question of how reaction conditions define accessible architecture. For wastewater-treatment materials, that distinction matters: the practical value of a morphology-control strategy depends not only on the final performance, but also on whether the route itself remains simple, scalable, and chemically clean enough to be useful beyond proof-of-concept demonstrations.

One representative structural outcome of this design logic is the formation of porous or mesoporous CeO_2_ frameworks under template-free hydrothermal conditions. In such systems, sequential precipitation, oxidation, and hydrothermal treatment can yield disordered mesoporous CeO_2_ with a type-IV(a) N_2_ adsorption–desorption isotherm, an H3 hysteresis loop, and pore-size characteristics consistent with open mesostructured frameworks [20]. These features are not merely textural descriptors. They indicate that the route can generate a host architecture combining internal pore volume, external accessibility, and relatively short diffusion pathways, all of which are directly relevant to dye uptake in aqueous media. In this sense, template-free porous CeO_2_ is significant not only because it avoids elaborate templating, but because it demonstrates that accessible adsorption-oriented architecture can be built through relatively simple synthetic control.

A second and conceptually complementary outcome is morphology evolution under template-free solvothermal conditions. In composition- or reagent-controlled CeO_2_ systems, systematic adjustment of the reaction environment can drive a sequence of structural changes ranging from compact nanodisks to nanoparticle aggregates and then to progressively developed dendritic architectures [21]. This type of result is important because it shows that morphology diversity in CeO_2_ can be generated through controlled precursor balance and growth competition rather than through externally imposed templating alone. In other words, template-free synthesis should not be understood as a simplified substitute for conventional structure-directed methods; it is a legitimate route to structurally differentiated oxide architectures with different accessibility, aggregation modes, and transport characteristics.

**Figure 2:**
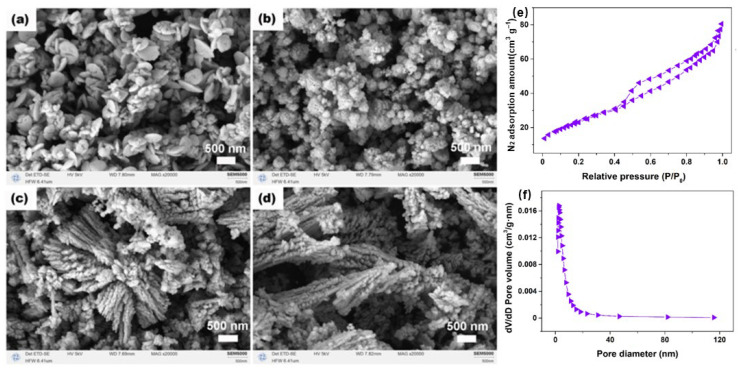
Route-controlled formation of accessible CeO_2_ architectures under template-free or low-template conditions. Panels (**a**–**d**) show morphology evolution from nanodisks to nanoparticle aggregates and then to dendritic structures with increasing Ce:N ratio in a solvothermal system. Panel (**e**) shows the N_2_ adsorption–desorption isotherm of porous CeO_2_, and panel (**f**) shows the corresponding pore-size distribution, indicating a disordered mesoporous framework (type IV(a) isotherm with H3 hysteresis loop; BET surface area 86.8 m^2^ g^−1^; pore volume 0.129 cm^3^ g^−1^; average pore size 6.2 nm). Combined from Refs. [21,22].

The architecture-forming significance of these routes is summarized in Figure 2. Panels (a–d) show that, under solvothermal control, CeO_2_ can evolve from compact nanodisk-like units to nanoparticle aggregates and then to more developed dendritic structures as the reaction environment changes. Panels (e) and (f) provide the complementary porosity evidence for a porous CeO_2_ host, showing a type IV(a) N_2_ adsorption–desorption isotherm with an H3 hysteresis loop and a pore-size distribution in the mesopore range. The reported BET surface area, pore volume, and average pore size are 86.8 m^2^ g^−1^, 0.129 cm^3^ g^−1^, and 6.2 nm, respectively [22]. Read together, these panels show that low-template or template-free processing in CeO_2_ does not merely generate different external morphologies, but can also define the accessible mesoporous framework through which dye molecules enter, diffuse, and interact with the host.

This point deserves emphasis because the most useful classification of CeO_2_ synthesis routes for dye-removal applications is not the conventional division into hydrothermal, solvothermal, precipitation, or oxidation-assisted methods taken separately. A more meaningful classification is to ask what kind of structure each route produces and how directly that structure serves aqueous pollutant removal. From that perspective, at least three architecture-forming tendencies can already be identified. The first is the generation of porous or mesoporous CeO_2_ frameworks with rapid dye uptake and short adsorption–desorption equilibration times [19]. The second is the formation of multilayered or nanoflake-assembled structures through solvothermal processing, which create open void space while maintaining crystallographically coherent CeO_2_ [19,22,23,24]. The third is the development of hierarchically assembled flower-like microspheres, where larger-scale structural organization is built from thinner subunits and can later be combined with low-level doping or optical tuning [19,25]. These route–structure correspondences provide a more useful way to read the CeO_2_ dye-removal literature than a simple list of synthesis methods.

Another important implication is that template-free synthesis should not be equated with structural simplicity. On the contrary, many of the most interesting CeO_2_ architectures in dye-removal studies emerge precisely from self-organization during hydrothermal or solvothermal treatment. Broad studies on ceria nanomaterials have already shown that hydrothermal methods can yield shape-dependent nanoparticles, self-assembled flower-like microspheres, and mesoporous structures through careful adjustment of solvent environment and precursor evolution [17,18,19]. The dye-removal-oriented literature extends this point by showing that porous CeO_2_ for fast adsorption, multilayered CeO_2_ for adsorption-assisted photocatalysis, and flower-like CeO_2_ for accelerated short-time uptake can all be obtained from routes that remain comparatively simple in formulation while still generating non-trivial structure. This is precisely why template-free and low-template CeO_2_ synthesis deserves attention at the level of architecture design rather than being treated as a purely preparative convenience.

From the standpoint of the present review, the most important conclusion is therefore not that one route is universally superior to another, but that route selection should be judged by the architecture it makes accessible. For CeO_2_ in dye-wastewater purification, the relevant structural outcomes are those that maximize accessible surface area, shorten diffusion pathways, facilitate transport within open frameworks, and, when needed, support local enrichment of dye molecules prior to light-assisted transformation. Once route is understood as a means of building porous, mesoporous, layered, or flower-like CeO_2_ architectures, the next question becomes how those architectures actually govern adsorption kinetics, equilibrium capacity, and removal efficiency in dye-containing aqueous systems. At this point, it is also important to distinguish larger-scale architecture from facet-level morphology. Shape-controlled CeO_2_ nanostructures such as nanorods, nanocubes, and nanooctahedra are not merely geometric variants of the same oxide; they differ in the relative exposure of crystal planes such as {111}, {100}, and {110}, which in turn affects surface energy, oxygen-vacancy formation tendency, adsorption energetics, and photocatalytic behavior. In this sense, facet-dependent surface chemistry represents a finer structural scale than the porous, multilayered, or flower-like architectures emphasized in the present review, but not a separate one. A more complete morphology-centered interpretation of CeO_2_ therefore needs to connect these two levels: larger-scale architecture determines accessibility and transport, whereas facet-level structure helps determine how the exposed CeO_2_ surface behaves once dye molecules arrive there.

## 3. Morphology–Adsorption Relationships: Porosity, Surface Accessibility, and Dye Uptake

If the previous section establishes that template-free and low-template routes can generate structurally diverse CeO_2_ architectures, the next question is what those architectures actually do in dye-containing aqueous systems. The available evidence suggests that morphology affects adsorption in at least three coupled ways: it determines how many surface sites are accessible to dye molecules, how easily those molecules can diffuse through the particle assembly, and how effectively the local structure can retain dye species long enough for adsorption equilibrium to be established [26,27,28,29,30]. In this sense, morphology in CeO_2_ is not a cosmetic descriptor but a functional variable that directly shapes uptake kinetics and removal efficiency.

A representative manifestation of this effect is found in porous and mesoporous CeO_2_ hosts, where accessible internal pore space and relatively short transport paths can support both rapid dye uptake and substantial equilibrium loading. In template-free porous CeO_2_ systems, the adsorption–desorption isotherm, pore-size distribution, and short-time removal behavior together indicate that the structural value of porosity lies not simply in creating more surface area but in organizing how dye molecules enter, diffuse through, and remain within the host architecture [20]. This point is especially important in aqueous dye removal, where the timescale of adsorption can be just as relevant as the final loading capacity. A porous CeO_2_ framework is therefore significant not only because it provides more accessible surface but because it can integrate pore volume, molecular transport, and adsorption-site exposure into a single host structure.

This interpretation becomes clearer when adsorption performance is considered together with structural accessibility rather than with BET surface area alone. In porous CeO_2_ systems, rapid AO7 uptake within the first tens of minutes, together with substantial Langmuir-type adsorption capacity and good recyclability, suggests that the relevant structural advantage is not simply high area in an abstract sense, but a pore architecture compatible with fast access, sufficient retention, and repeated use [30,31,32,33]. This distinction matters because it prevents the discussion from collapsing into a purely textural interpretation. In morphology-controlled CeO_2_, adsorption is governed not by area alone, but by how that area is distributed and whether it is available along realistic transport pathways.

A second and complementary picture emerges from layered and flower-like CeO_2_ architectures. Here, the structural advantage is not necessarily maximum pore volume or the highest absolute adsorption capacity, but the presence of open interleaved frameworks that allow dye molecules to reach surface-active sites quickly. In flower-like CeO_2_ systems, adsorption–desorption equilibrium can be established on a much shorter timescale than in less open architectures, even when the final equilibrium capacity remains more moderate than that of highly porous hosts [26,27,28,29]. This kind of behavior is analytically important because it shows that morphology–adsorption relationships in CeO_2_ are not one-dimensional. Different architectures can favor different aspects of adsorption performance: porous mesostructures may prioritize accessible internal loading, whereas layered flower-like assemblies may prioritize short-time uptake and rapid equilibration.

The interfacial chemistry proposed for flower-like CeO_2_ supports the same broader conclusion. In these systems, adsorption is associated with protonation of surface hydroxyl groups to form positively charged Ce–OH^2+^ species, electrostatic attraction between these sites and the sulfonate groups of anionic dyes, and possible bidentate interaction between surface cerium centers and sulfonate moieties [20]. Because the protonation state of surface hydroxyl groups depends on solution pH relative to the isoelectric point of CeO_2_, this electrostatic pathway should be understood as condition-dependent rather than universal. The significance of this mechanism is not merely chemical. It shows that morphology governs more than the number of sites; it governs whether such sites remain exposed and accessible in aqueous solution. A layered or flower-like architecture can therefore be advantageous not simply because it looks hierarchical, but because it helps preserve effective contact between dye molecules and chemically relevant surface configurations.

**Figure 3:**
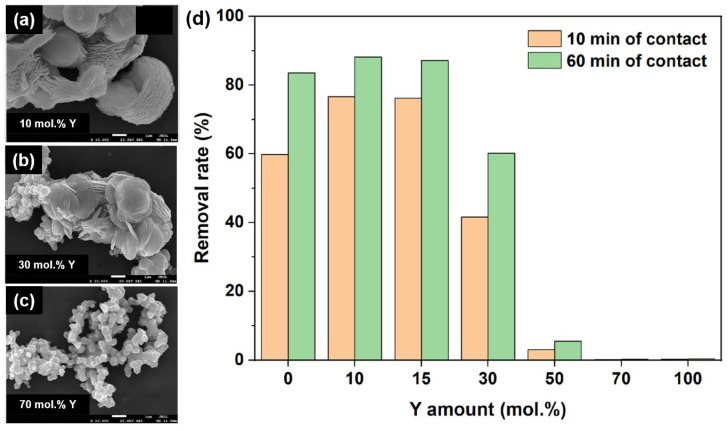
Morphology-dependent adsorption behavior in Y-modified flower-like CeO_2_. Panels (**a**–**c**) show representative morphologies of CeO_2_ synthesized with 10 mol.% Y, 30 mol.% Y, and 70 mol.% Y, respectively, highlighting the transition from a retained multilayered host to coral-like aggregate structures at higher Y input. Panel (**d**) compares AO7 removal rates at 10 and 60 min for the corresponding samples, showing that adsorption performance changes with morphology evolution rather than increasing monotonically with Y addition. Adapted from Ref. [20].

The morphology-dependent adsorption behavior discussed above is summarized more directly in Figure 3. Panels (a–c) compare representative morphologies obtained at 10, 30, and 70 mol.% Y additions, respectively, showing that the multilayered flower-like host is largely retained at low Y input but progressively transforms toward coral-like aggregate structures as the Y amount increases. Panel (d) compares AO7 removal rates at 10 and 60 min for the corresponding samples and shows that adsorption performance does not increase monotonically with Y addition. Instead, low-level Y incorporation is associated with improved short-time uptake and high near-equilibrium removal, whereas excessive Y input accompanies morphological collapse and severe deterioration in adsorption behavior. In this sense, Figure 3 is not intended to imply that composition alone determines adsorption; rather, it shows that adsorption performance changes together with host-structure evolution, so that morphology and adsorption must be interpreted in a coupled way rather than as independent variables [20]. For the Y-doped flower-like CeO_2_ system, the available textural data further support this interpretation. The reported specific surface areas of undoped, 2 mol.%, 4 mol.%, and 9 mol.% Y-doped CeO_2_ were 96.0, 102.0, 98.1, and 98.4 m^2^ g^−1^, respectively, indicating only minor variation in BET surface area across the adsorption-relevant low-doping regime. This result is important because it shows that the improved short-time uptake and equilibrium behavior in the optimally doped samples cannot be attributed simply to a larger surface area, but must instead be interpreted together with the morphology-retention effect and the accompanying changes in local surface chemistry and defect structure [20].

Taken together, these cases suggest that morphology–adsorption relationships in CeO_2_ are best understood through three linked descriptors: accessible porosity, architecture-dependent transport, and exposure of chemically relevant surface sites. Porous and mesoporous CeO_2_ architectures favor rapid uptake and high equilibrium loading because they provide both internal pore volume and short transport pathways [21,22,26,30]. Layered and flower-like assemblies, in contrast, may operate with more modest absolute capacities but can still achieve very fast equilibration and high short-time removal efficiency because their open interleaved structures facilitate dye access to exposed Ce-centered adsorption sites [20,23,27,28,29]. In both cases, the key issue is not morphology in a merely geometric sense but morphology as an organizer of mass transfer and interfacial interaction.

From the standpoint of the present review, the most important conclusion is therefore that adsorption performance in CeO_2_-based dye-removal systems should not be interpreted through surface area alone. BET values, Langmuir capacities, and equilibrium times remain useful, but they become mechanistically meaningful only when read together with pore architecture, particle assembly, and surface-interaction pathways [30,31,32]. The porous CeO_2_ case shows what happens when a template-free route creates a mesostructure optimized for rapid uptake and high loading; the flower-like CeO_2_ case shows how layered hierarchical assemblies can accelerate adsorption even when capacity gains are moderate; and the comparison highlighted in Figure 3 makes clear that morphology is not a secondary descriptor but one of the primary determinants of dye uptake in CeO_2_-based systems. Once this is recognized, the next question becomes how adsorption can be coupled with light-driven transformation in the same structural platform.

## 4. Beyond Adsorption: Coupled Adsorption–Photocatalytic Removal in CeO_2_ Systems

Adsorption is often the first function discussed in CeO_2_-based dye-removal studies, but it is not always the final one. Once a suitable architecture has enriched dye molecules at the catalyst surface, CeO_2_ can also operate as a light-responsive oxide in which adsorption and photocatalytic transformation become sequentially coupled rather than conceptually separate. This distinction is important. If morphology determines how efficiently dye molecules are captured and held near the surface, then the next design question is whether that same structural platform can also support their subsequent degradation under irradiation. From this perspective, adsorption should not be treated as a competing explanation for photocatalysis, but as the step that often conditions whether photocatalytic removal can proceed efficiently at all.

A representative case is found in multilayered Pr-doped CeO_2_ systems designed for simultaneous adsorption and photocatalytic degradation of Acid Orange 7. In such materials, the fluorite structure is retained after Pr incorporation, while the host preserves an open multilayered mesoporous framework capable of capturing dye molecules before irradiation [23]. This synthesis–structure combination is especially instructive because it shows that photocatalytic function does not replace the morphology-centered logic established in the preceding sections. Instead, light-assisted removal is built on top of an already adsorption-active CeO_2_ host.

The most important mechanistic point is that dark adsorption and light-driven degradation are experimentally distinguishable but functionally linked. Under dark conditions, adsorption–desorption equilibrium is established within a relatively short period, confirming that adsorption remains the first event even in a photocatalytic experiment [23]. Once ultraviolet irradiation is introduced, the removal rate increases sharply, particularly in the optimally doped regime. This sequence is significant because it shows that the catalyst does not simply degrade dissolved dye molecules directly from bulk solution. Rather, dye molecules are first captured and enriched near the surface, and only then does the structured CeO_2_ host, together with doping-modified optical and defect properties, promote further transformation under irradiation. In this sense, adsorption and photocatalysis in CeO_2_ systems are not parallel and independent contributions, but sequentially linked processes.

This interpretation is also more useful than the oversimplified statement that more vacancies necessarily give better photocatalysis. In representative Pr-doped CeO_2_ systems, the factors controlling dye removal are not single, but include grain size, oxygen-vacancy defects, optical response, and surface accessibility [33,34,35,36,37]. Even in a section devoted to photocatalytic removal, morphology and structure therefore cannot be set aside. Grain refinement changes how easily dye molecules approach the surface; vacancy defects alter charge trapping and reactive oxygen generation; optical darkening and reflectance changes affect light harvesting; and the multilayered mesoporous host still determines whether dye molecules can be enriched at the active interface before irradiation begins. The important point is not that photocatalysis supersedes adsorption, but that photocatalysis becomes effective only after adsorption has already organized the local reaction environment.

**Figure 4:**
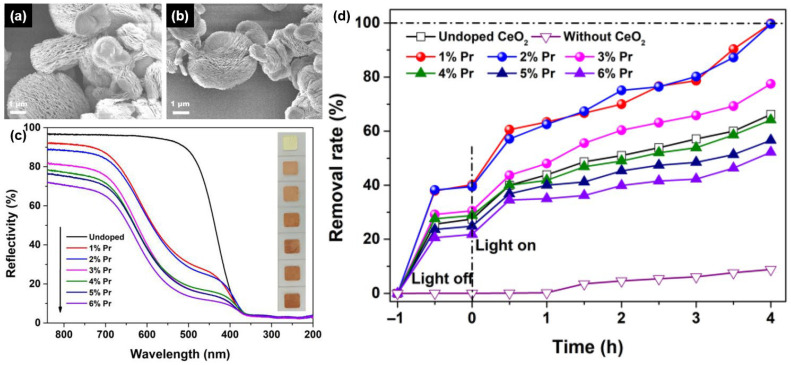
Adsorption-assisted photocatalytic removal in morphology-retained Pr-doped CeO_2_. Panels (**a**,**b**) show that undoped and Pr-doped CeO_2_ retain a multilayered porous host morphology after doping, respectively. Panel (**c**) presents the reflectance spectra and visible color evolution of undoped and Pr-doped CeO_2_, indicating doping-induced optical-response changes. Panel (**d**) compares dark adsorption and ultraviolet-light-assisted AO7 removal, showing that adsorption occurs first and that low-level Pr doping enhances subsequent photocatalytic degradation. The ultraviolet irradiation in panel (**d**) was provided by a medical ultraviolet lamp (300 W, λ = 254 nm). Adapted from Ref. [23].

This transition from adsorption to adsorption-assisted photocatalysis is summarized in Figure 4. Panels (a) and (b) show that undoped and Pr-doped CeO_2_ retain a multilayered porous host morphology after doping, respectively, indicating that the photocatalytic system is built on a morphology-retained host rather than on a newly created architecture. Panel (c) shows the reflectance response and visible color evolution of undoped and Pr-doped CeO_2_, consistent with a doping-induced change in optical behavior. Stoichiometric CeO_2_ typically exhibits a band gap of about 3.0–3.2 eV, so the reflectance changes induced by Pr doping are interpreted here as a modification of optical response and absorption-edge behavior rather than as the formation of a fundamentally different host architecture. The functional consequence of this defect- and optical-response modification is also quantitative rather than merely qualitative: in the representative Pr-doped system, dark adsorption increased from 25.6% for undoped CeO_2_ to 37.9% and 38.2% for 1 and 2 mol.% Pr-doped samples, respectively, while the final AO7 removal after 4 h of ultraviolet irradiation increased from 66.2% for undoped CeO_2_ to nearly complete decolorization in the optimally doped samples [23]. Panel (d) compares dark adsorption and UV-assisted AO7 removal and shows that adsorption occurs first, while low-level Pr doping strengthens the subsequent light-assisted degradation step under ultraviolet irradiation. Read in this way, Figure 4 supports a sequential interpretation: dye molecules are first captured and enriched by the structured CeO_2_ host, and only then does the optical and redox response of the doped system promote further transformation under irradiation [22].

The proposed radical-based mechanism supports the same sequence. Under ultraviolet irradiation, electrons are excited from the valence band to the conduction band of doped CeO_2_, leaving holes in the valence band [23,33,34,35,36,37]. These charge carriers then react with adsorbed water and oxygen to form oxidative species such as ·OH and ·O^2−^, while hydrogen peroxide and active oxygen species may also participate in subsequent transformation steps. The significance of doping here is therefore twofold: it modifies the optical response of the host and it changes the defect landscape through oxygen-vacancy generation, thereby influencing charge separation and reactive-species formation. At the same time, these systems also make clear that adsorption-assisted photocatalysis requires balance. If the surface is excessively covered by dye molecules, light access to the catalyst and subsequent surface reactions can be hindered. The catalyst must therefore adsorb enough dye to ensure local enrichment, but its architecture must also preserve sufficient surface exposure for light absorption and redox turnover.

This balance helps explain why not every CeO_2_ adsorbent automatically becomes an efficient photocatalyst. Porous CeO_2_ hosts optimized mainly for rapid uptake and high equilibrium loading are not necessarily optimal photocatalysts, because adsorption performance and light-driven transformation do not respond to exactly the same structural requirements [21,23]. In contrast, adsorption-assisted photocatalytic systems rely on a more carefully balanced architecture in which capture, retention, light harvesting, and reactive-species generation remain compatible. The difference is not simply one of composition, but of functional orientation. In one case, structure is tuned primarily to maximize adsorption; in the other, structure is tuned so that adsorption can be followed by effective surface-mediated transformation under irradiation.

From the standpoint of the present review, the most important conclusion is therefore that adsorption–photocatalysis coupling in CeO_2_ systems should be interpreted as a structure-governed sequence. Morphology first determines whether dye molecules can be captured efficiently and held in proximity to the CeO_2_ surface. Optical response, defect states, and redox behavior then determine whether those adsorbed molecules can be transformed further under irradiation. Once this is recognized, the role of doping in this review becomes easier to define. Doping is not the primary design axis here in the same way it was in the oxygen-storage review. Instead, it functions as a secondary tuning strategy that can reinforce a morphology-centered removal platform by adjusting adsorption capability, light absorption, and reactive-species generation within an already favorable architecture.

## 5. When Doping Serves Morphology-Controlled Removal Rather than Oxygen Storage

In the present review, doping should not be treated as the primary design axis in the same way it is often treated in studies of oxygen-storage ceria. Here, the more relevant question is not how dopants regulate oxygen vacancies for oxygen buffering, but how they refine an already favorable morphology-centered platform for dye removal. In aqueous dye systems, porous accessibility, hierarchical assembly, and exposed surface-active sites usually determine whether CeO_2_ can adsorb pollutants rapidly and efficiently in the first place. Only after such a host architecture has been established does doping become meaningful as a secondary variable that can strengthen adsorption kinetics, alter surface interaction, broaden light absorption, or improve adsorption-assisted photocatalytic removal [21,24]. This distinction is important because it keeps the present review conceptually separate from a vacancy-centered or oxygen-storage-centered discussion of doped ceria.

At the same time, a morphology-centered interpretation should not be read as a morphology-only interpretation. In CeO_2_ systems, host architecture and surface/defect chemistry are coupled rather than independent. The Ce^3+^/Ce^4+^ redox couple, oxygen-vacancy population, local adsorption energetics, optical absorption behavior, and charge-carrier separation or recombination all influence whether a given CeO_2_ host functions only as an adsorbent or further as an adsorption-assisted photocatalyst. Morphology remains important because it defines the host within which these processes occur, but the resulting performance can only be understood fully when architecture is read together with surface chemistry and defect chemistry.

A useful way to frame this issue is to distinguish between host construction and host refinement. In morphology-controlled dye-removal systems, the host is created first through synthesis route and structural assembly. Porous, multilayered, or flower-like CeO_2_ architectures define the accessible pore space, transport pathways, and exposure of adsorption-relevant surface sites. Doping then becomes important only insofar as it modifies the behavior of that host without destroying the structural features that made it effective in the first place. In this sense, doping in CeO_2_-based dye-removal systems is most reasonably understood as a refinement strategy rather than a host-defining strategy. This logic is particularly clear in morphology-retained rare-earth-doped CeO_2_ systems. In multilayered Pr-doped CeO_2_, rare-earth incorporation changes the optical response, defect landscape, and dark adsorption behavior while preserving the fluorite framework and the broader porous layered host [33,34,35,36,37]. In flower-like Y-doped CeO_2_, low-level substitution similarly improves short-time dye uptake and equilibrium capacity while retaining the hierarchical architecture that enables fast mass transfer and open access to surface adsorption sites [26,27,28,29]. These two cases are structurally different, but they converge on the same general point: doping improves what an already well-formed host can do; it does not create the essential host architecture itself.

**Figure 5:**
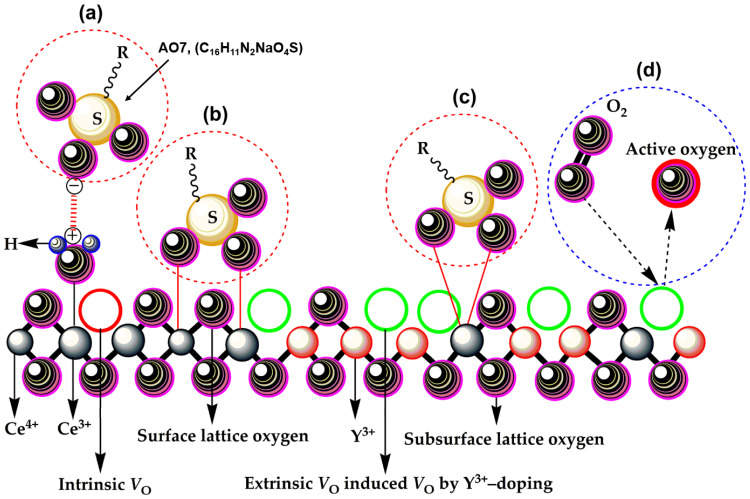
Proposed adsorption mechanism of Acid Orange 7 (AO7) on Y-doped flower-like CeO_2_. Panel (**a**) illustrates electrostatic attraction between protonated surface hydroxyl groups and the anionic sulfonate group of AO7. Panels (**b**,**c**) show possible bidentate interactions between the sulfonate group and surface Ce cations. Panel (**d**) shows the possible involvement of vacancy-associated active oxygen species. This figure is used here to clarify how local surface chemistry refines the function of a morphology-defined CeO_2_ host. Adapted from Ref. [20].

This distinction becomes especially important when adsorption performance is interpreted mechanistically. In morphology-centered CeO_2_ systems, the improvement associated with doping cannot be reduced to a simple increase in BET surface area. In the representative cases discussed here, rare-earth incorporation either changes the specific surface area only modestly or leaves the broader host morphology essentially intact, yet still produces measurable gains in adsorption kinetics, short-time removal efficiency, or coupled adsorption–photocatalytic performance [20,23]. Such behavior indicates that doping acts mainly by changing local surface chemistry, defect structure, optical response, and the reactivity of adsorption-relevant sites, rather than by replacing architecture-level control with composition-level control. The surface-chemistry aspect of morphology-controlled adsorption is summarized in Figure 5. Panel (a) illustrates electrostatic attraction between protonated surface hydroxyl groups and the anionic sulfonate group of AO7, while panels (b) and (c) show possible bidentate interaction pathways between sulfonate groups and surface Ce centers. Panel (d) further indicates the possible involvement of vacancy-associated active oxygen species. Taken together, these pathways clarify that the adsorption behavior of flower-like CeO_2_ cannot be interpreted only in geometric terms. The hierarchical host is important because it preserves access to surface-active sites, but the resulting adsorption response also depends on how those sites interact chemically with dye molecules at the interface. In this sense, Figure 5 complements the morphology discussion by showing how local surface chemistry can refine the function of a morphology-defined CeO_2_ host [20]. The adsorption pathways discussed above should also be interpreted together with the pH dependence of the CeO_2_ surface. The surface charge of CeO_2_ is not fixed, but varies with solution pH relative to its isoelectric point. Below the isoelectric point, protonation of surface hydroxyl groups favors electrostatic attraction toward anionic sulfonate-containing dyes such as AO7, whereas this interaction is expected to weaken as the surface becomes less positively charged at higher pH. The adsorption mechanism of AO7 on CeO_2_ should therefore be understood as the combined result of host architecture, site accessibility, and pH-dependent surface chemistry rather than as a purely geometric or purely electrostatic process. The isoelectric point of CeO_2_ is commonly reported in the near-neutral range (typically around pH 6–8, depending on synthesis history and surface hydroxylation), so protonation-assisted adsorption of anionic AO7 is more favorable under mildly acidic conditions than under alkaline conditions.

Seen in this way, the hierarchy of design in CeO_2_-based dye removal becomes clearer. First, synthesis route determines whether CeO_2_ develops into a porous, mesoporous, layered, or flower-like host architecture [20,21,22,23,24,25,38]. Second, that architecture governs adsorption-site accessibility, diffusion pathways, and the local enrichment of dye molecules [26,27,28,29,30,31,32]. Third, only then does doping become useful, either by strengthening adsorption through local surface or defect-mediated effects, or by coupling adsorption with improved optical and redox behavior under irradiation [23,33,34,35,36,37]. This hierarchy matters because it prevents the discussion from drifting back into the logic of the oxygen-storage review. In the first review, doping itself is the central problem. Here, morphology remains the first-order variable, and doping is best understood as a secondary strategy that helps a favorable host structure perform better. This perspective also explains why not all doped CeO_2_ systems are equally relevant to the present article. Many doped-ceria studies focus primarily on redox chemistry, oxygen vacancies, visible-light extension, or catalytic oxidation under conditions where morphology is not the central analytical variable. Such studies may be important in their own right, but they are not automatically informative for morphology-controlled dye removal. The systems most relevant here are those in which the host structure is clearly established first and doping then modifies adsorption behavior, light-response characteristics, or the efficiency of adsorption-assisted transformation without erasing the structural identity of the host. This is precisely why low-level rare-earth-doped multilayered and flower-like CeO_2_ architectures are so useful for the present review: they show that composition tuning can be meaningful without displacing the primary importance of route-controlled architecture.

From the standpoint of the present review, the most important conclusion is therefore that doping in CeO_2_-based dye-removal systems should be framed as structure-assisted tuning rather than as the primary design principle. The porous or hierarchical host still determines whether pollutants can reach and occupy the CeO_2_ surface efficiently. Doping then becomes valuable insofar as it strengthens adsorption-site chemistry, improves uptake kinetics, broadens optical response, or increases the efficiency of light-assisted degradation without compromising the morphology that made the system effective in the first place. Once route and morphology are recognized as the primary determinants of CeO_2_-based dye removal, and doping is repositioned as a secondary but useful modifier, the final question becomes how these insights should shape future design priorities for wastewater-treatment materials.

A morphology-centered discussion of CeO_2_ for wastewater purification also remains incomplete without considering material stability in aqueous environments. Although many published studies report promising short-term adsorption performance and, in some cases, several reuse cycles, the current literature is still less systematic in evaluating cerium leaching under different pH conditions, structural stability of porous or hierarchical hosts in water, and possible surface evolution under ultraviolet irradiation. In adsorption-assisted photocatalytic systems, repeated irradiation may alter surface hydroxylation, vacancy populations, or dye-accessible interfaces even when the bulk fluorite structure remains intact. For this reason, reported recyclability should not be treated as equivalent to long-term practical stability. For example, a representative porous CeO_2_ adsorbent retained more than 92.5% of its AO7 removal efficiency after five adsorption–desorption cycles, which is encouraging but still illustrates that much of the present literature remains limited to short-cycle stability assessment rather than true long-term durability evaluation [21]. Quantitative Ce-leaching data under dye-removal conditions remain comparatively scarce, and this lack of systematic reporting itself underscores the need for more rigorous aqueous-stability studies. Future work should place greater emphasis on regeneration efficiency, retention of host architecture over multiple cycles, possible Ce release into aqueous media, and durability under more realistic operating conditions.

## 6. Conclusions and Outlook

The central argument of this mini-review is that CeO_2_-based dye-wastewater purification is best understood through a morphology-centered design logic rather than through isolated composition changes. Across the systems discussed above, the most durable lesson is not that one specific composition always performs best, but that synthesis route determines architecture, architecture governs adsorption and local enrichment, and only on that basis can additional functions such as light-assisted degradation or compositional tuning become effective. In this sense, the key scientific problem is not simply how to make CeO_2_ more active but how to construct a host architecture in which accessibility, transport, and surface reactivity are favorably coordinated. Several conclusions follow from this perspective. First, template-free and low-template routes are important not merely because they simplify synthesis, but because they can generate structurally meaningful CeO_2_ hosts, including porous, mesoporous, multilayered, and flower-like architectures. These structures differ not only in appearance, but in how they organize pore accessibility, diffusion pathways, and exposure of adsorption-relevant surface sites. Second, adsorption in CeO_2_-based dye-removal systems should not be treated as a secondary or preliminary phenomenon. It is the structural first step that determines whether dye molecules can be captured efficiently and enriched near the active surface. Third, doping in these systems is most useful when it refines an already favorable host architecture by modifying local surface chemistry, optical response, or reactive-species generation, rather than replacing the central importance of morphology.

This view also clarifies why the present review differs fundamentally from a defect- or oxygen-storage-centered discussion of doped ceria. In dye-wastewater purification, the primary design variable is not vacancy chemistry by itself, but the architecture that determines how pollutants access the CeO_2_ surface and how long they remain in contact with adsorption- or transformation-relevant sites. Defect states still matter, especially when adsorption is coupled with photocatalytic removal, but their function is subordinate to the broader route–architecture–function sequence. Put differently, morphology defines the host; defect and optical effects refine what that host can do.

The most useful direction for future work is therefore to move from morphology description to morphology–function prediction. Much of the current literature can already show that porous or hierarchical CeO_2_ performs well in dye removal, but the field is still less effective at specifying which structural descriptors matter most and why. Future progress will depend on the more explicit correlation of architecture with accessible porosity, transport path length, subunit assembly, surface-site exposure, and regeneration behavior. The goal should not simply be to report another successful morphology, but to identify what kinds of host structure consistently favor rapid uptake, robust equilibrium capacity, and stable cycling performance. A second priority is to treat adsorption and photocatalysis as a coupled sequence rather than as competing explanations. In many CeO_2_-based systems, adsorption determines whether dye molecules are first localized at the surface, while optical and redox processes determine whether those adsorbed species can then be transformed further under irradiation. Future design should therefore aim at balanced platforms in which dye enrichment, light harvesting, charge separation, and reactive-species generation remain compatible with one another. Optimizing only one of these functions in isolation is unlikely to provide the most effective overall removal system. From a design standpoint, porous or mesoporous CeO_2_ hosts are most suitable when rapid uptake and high equilibrium loading are prioritized, because they combine accessible pore volume with relatively short transport pathways. Multilayered or flower-like assemblies are more advantageous when fast equilibration and exposed surface-active sites are especially important. Morphology-retained doped CeO_2_ becomes most useful when adsorption must be coupled with photocatalytic transformation, since doping can refine optical response, defect chemistry, and surface reactivity without necessarily replacing a favorable host structure. In practical terms, the most effective strategy is therefore not to treat morphology control and doping as competing design routes but to combine them hierarchically, with host construction first and secondary tuning afterward. A third priority concerns practical relevance. Many current studies still rely on model dyes and relatively idealized single-solute conditions. These are useful for establishing structure–performance relationships, but they are not sufficient for judging real applicability. Because much of the currently available literature still relies on Acid Orange 7 as an anionic model dye, the generality of the morphology–adsorption–photocatalysis relationships proposed here should be tested further across cationic dyes, dyes with different molecular dimensions, mixed-dye systems, and more complex wastewater matrices. More realistic testing under mixed-solute conditions, varying ionic strength, pH fluctuation, and repeated regeneration will be necessary if morphology-engineered CeO_2_ is to move beyond proof-of-concept demonstrations. In this regard, the most valuable future studies are likely to be those that combine structural control with practical durability rather than those that focus on short-term performance alone.

More broadly, the significance of CeO_2_ in dye-wastewater purification lies in the fact that it is not merely another oxide adsorbent or photocatalyst. It is a structurally programmable platform. Once this is recognized, the design problem becomes clearer: not which single modification is universally best, but how route, architecture, adsorption behavior, and secondary tuning can be combined to create integrated removal platforms with predictable function. That shift—from empirical performance optimization to deliberate structure-directed design—is likely to define the next stage of progress in CeO_2_-based wastewater purification.

## Data Availability

No new data were created or analyzed in this study. Data sharing is not applicable.
